# Comparison of Soluble Guanylate Cyclase Stimulators and Activators in Models of Cardiovascular Disease Associated with Oxidative Stress

**DOI:** 10.3389/fphar.2012.00128

**Published:** 2012-07-05

**Authors:** Melissa H. Costell, Nicolas Ancellin, Roberta E. Bernard, Shufang Zhao, John J. Upson, Lisa A. Morgan, Kristeen Maniscalco, Alan R. Olzinski, Victoria L. T. Ballard, Kenny Herry, Pascal Grondin, Nerina Dodic, Olivier Mirguet, Anne Bouillot, Francoise Gellibert, Robert W. Coatney, John J. Lepore, Beat M. Jucker, Larry J. Jolivette, Robert N. Willette, Christine G. Schnackenberg, David J. Behm

**Affiliations:** ^1^Heart Failure Discovery Performance Unit, Metabolic Pathways and Cardiovascular Therapy Area Unit, GlaxoSmithKlineKing of Prussia, PA, USA; ^2^Lipid Metabolism Discovery Performance Unit, Metabolic Pathways and Cardiovascular Therapy Area UnitGlaxoSmithKline, Les Ulis, France

**Keywords:** soluble guanylate cyclase, cGMP, BAY 60-4552, GSK2181236A, VASP, SHR-SP

## Abstract

Soluble guanylate cyclase (sGC), the primary mediator of nitric oxide (NO) bioactivity, exists as reduced (NO-sensitive) and oxidized (NO-insensitive) forms. We tested the hypothesis that the cardiovascular protective effects of NO-insensitive sGC activation would be potentiated under conditions of oxidative stress compared to those of NO-sensitive sGC stimulation. The cardiovascular effects of the NO-insensitive sGC activator GSK2181236A [a low, non-depressor dose, and a high dose which lowered mean arterial pressure (MAP) by 5–10 mmHg] and those of equi-efficacious doses of the NO-sensitive sGC stimulator BAY 60-4552 were assessed in (1) Sprague Dawley rats during coronary artery ischemia/reperfusion (I/R) and (2) spontaneously hypertensive stroke prone rats (SHR-SP) on a high salt/fat diet (HSFD). In I/R, neither compound reduced infarct size 24 h after reperfusion. In SHR-SP, HSFD increased MAP, urine output, microalbuminuria, and mortality, caused left ventricular hypertrophy with preserved ejection fraction, and impaired endothelium-dependent vasorelaxation. The low dose of BAY 60-4552, but not that of GSK2181236A, decreased urine output, and improved survival. Conversely, the low dose of GSK2181236A, but not that of BAY 60-4552, attenuated the development of cardiac hypertrophy. The high doses of both compounds similarly attenuated cardiac hypertrophy and improved survival. In addition to these effects, the high dose of BAY 60-4552 reduced urine output and microalbuminuria and attenuated the increase in MAP to a greater extent than did GSK2181236A. Neither compound improved endothelium-dependent vasorelaxation. In SHR-SP isolated aorta, the vasodilatory responses to the NO-dependent compounds carbachol and sodium nitroprusside were attenuated by HSFD. In contrast, the vasodilatory responses to both GSK2181236A and BAY 60-4552 were unaltered by HSFD, indicating that reduced NO-bioavailability and not changes in the oxidative state of sGC is responsible for the vascular dysfunction. In summary, GSK2181236A and BAY 60-4552 provide partial benefit against hypertension-induced end-organ damage. The differential beneficial effects observed between these compounds could reflect tissue-specific changes in the oxidative state of sGC and might help direct the clinical development of these novel classes of therapeutic agents.

## Introduction

Nitric oxide (NO), a key endogenous signaling molecule best known for its robust vasodilator actions (Palmer et al., 1987; Furchgott, 1988), is involved in numerous biological processes (Pacher et al., 2007). Diminished release of nitric oxide because of impaired synthesis and/or excessive oxidative degradation plays an important role in the development of multiple cardiovascular diseases, including hypertension and heart failure (Evgenov et al., 2006; Bauersachs and Widder, 2008). As such, organic nitrates, known as NO-donors, have been utilized as cardiovascular therapeutics for over 140 years (Brunton, 1867; Lindenfeld et al., 2010). However, long-term nitrate usage is limited due to tolerance and increased NO scavenging under conditions of oxidative stress. Indeed, elevated levels of peroxynitrite, produced by the interaction between NO and superoxide anion, lead to increased cytotoxicity and can thus exacerbate disease progression (Pacher et al., 2007).

Soluble guanylate cyclase (sGC) is the primary NO receptor and mediates the formation of cyclic guanosine 3′,5′-monophosphate (cGMP), the secondary messenger compulsory for the vasodilatory response to NO. Small molecule drugs directly targeting sGC are currently being investigated as an alternative approach to nitrate therapy for increasing cGMP levels (Evgenov et al., 2006).

Two novel groups of small molecule compounds which increase the enzymatic activity of sGC have been identified over the past two decades, sGC “stimulators” and “activators” (Ko et al., 1994; Stasch et al., 2002b). The effectiveness of these compounds differs depending on the oxidation state of sGC. sGC is a heterodimeric heme-containing enzyme, consisting of α- and β-subunits. Like the endogenous ligand NO, sGC stimulators, including YC-1, BAY 41-2272, BAY 41-8543, BAY 60-4552, and BAY 63-2521 (riociguat), increase sGC activity only when the heme iron is in its reduced state (Fe^2+^; Ko et al., 1994; Stasch et al., 2002a; Evgenov et al., 2006). These compounds work synergistically with NO and are therefore considered “NO sensitizers.” Conversely, sGC activators, including BAY 58-2667 (cinaciguat), S3448, and HMR1766 (ataciguat), increase the activity of the enzyme only when the heme iron is oxidized (Fe^3+^) or the heme group is missing (Stasch et al., 2002b; Schindler et al., 2006). The effects of sGC activators are additive to NO.

Oxidative stress, a risk factor associated with various cardiovascular diseases including hypertension, atherosclerosis, diabetes, and heart failure, can result in oxidation and subsequent loss of the sGC heme, rendering the enzyme insensitive to either endogenous/exogenous NO or sGC stimulators (Mitrovic et al., 2011). As sGC activators preferentially bind to the enzyme in its oxidized or heme-free state, it has been proposed that this class of compounds might be advantageous in disease states associated with oxidative stress (Evgenov et al., 2006; Stasch et al., 2006). In support of this hypothesis, the *in vitro* vasodilatory effects of the sGC activator cinaciguat (but not the NO donor glycerol trinitrate) were potentiated under numerous pathological conditions associated with oxidative stress including atherosclerosis, hypertension, and type 2 diabetes (Stasch et al., 2006).

**Figure 1:**
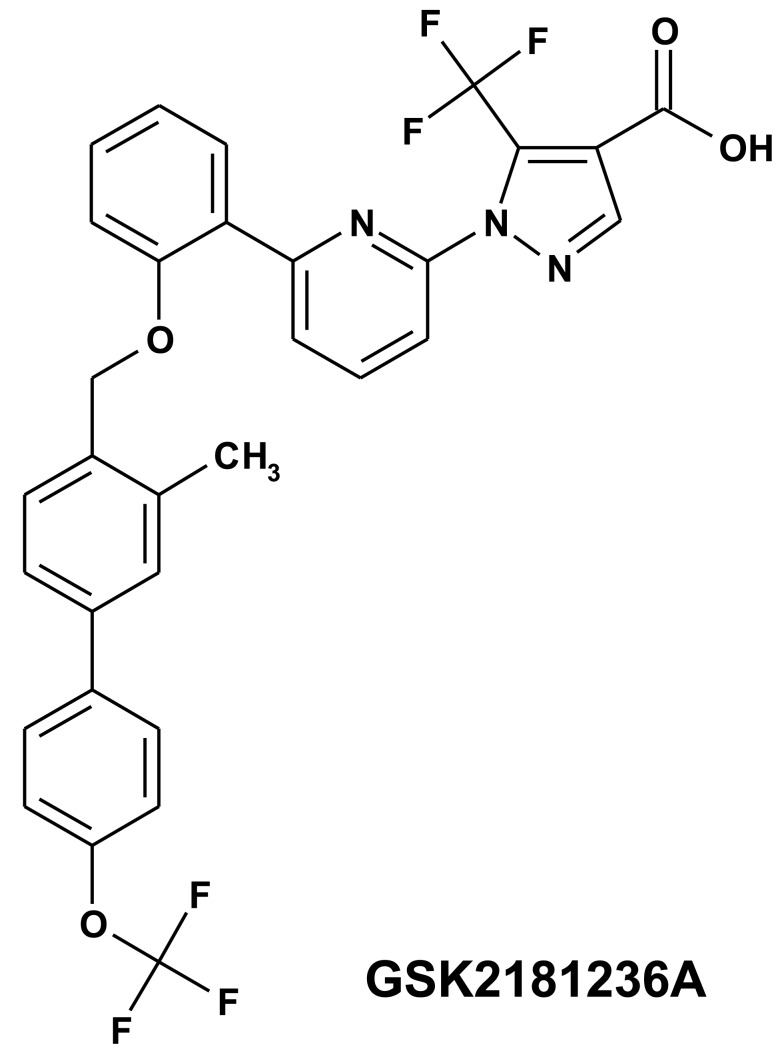
**Structure of the novel sGC activator GSK2181236A (1-(6-{2-[({3-methyl-4′-[(trifluoromethyl)oxy]-4-biphenylyl}methyl)oxy]phenyl}-2-pyridinyl)-5-(trifluoromethyl)-1H-pyrazole-4-carboxylic acid)**.

Although sGC activators are expected to be advantageous over sGC stimulators in disease states associated with oxidative stress, to our knowledge, this hypothesis has never been comprehensively tested *in vivo*. As such, in the present study, we compared the *in vivo* effects of the sGC stimulator BAY 60-4552 (Mitrovic et al., 2009) with a newly identified sGC activator, GSK2181236A (Figure 1) on cardiovascular physiology. Focus was placed on acute and chronic models of cardiovascular disease associated with oxidative stress: (1) acute coronary artery ischemia/reperfusion (I/R) in normotensive rats and (2) chronic high salt/fat diet (HSFD) in spontaneously hypertensive stroke prone rats (SHR-SP). Whereas neither compound attenuated cardiac I/R injury, both compounds improved survival and provided partial protection against chronic HSFD-induced end-organ damage. Interestingly, however, only sGC activation attenuated cardiac hypertrophy in a blood pressure-independent manner. In addition, in comparison to the blunted vasodilatory responses to the NO-dependent vasodilators carbachol and sodium nitroprusside (SNP), the vasodilatory effects of both the sGC stimulator and activator were not altered by chronic HSFD in SHR-SP. Overall, these results do not support the hypothesis that activation of NO-insensitive sGC provides a greater beneficial effect on cardiovascular hemodynamics, including blood pressure, heart rate, vascular resistance, and end-organ damage, than does NO-sensitive sGC stimulation, but suggest that sGC activation might be advantageous over sGC stimulation for mitigating cardiac hypertrophy associated with cardiovascular disease.

## Materials and Methods

All animal studies were performed in Association for Assessment and Accreditation of Laboratory Animal Care-accredited facilities in accordance with the Guide for Care and Use of Laboratory Animals (NIH Publication 85-23). Experimental protocols were reviewed and approved by the respective Institutional Animal Care and Use Committee at GlaxoSmithKline and CV Dynamics, Inc. (Newark, NJ, USA).

### Phosphorylation of vasodilator-stimulated phosphoprotein

The potency, efficacy, and mechanism of action of the sGC modulators GSK2181236A and BAY 60-4552 were assessed by measuring phosphorylation of the protein kinase G (PKG) substrate vasodilator-stimulator protein (VASP) in rat aortic smooth muscle cells. Briefly, in 96-well tissue culture test plates (Techno Plastic Products, Trasadingen, Switzerland), primary rat aortic smooth muscle cells were incubated at 37°C for 10 min in the presence or absence of 10 μM 1H-[1,2,4]oxadiazolo[4,3-a]quinoxalin-1-one (ODQ), a highly selective, and irreversible sGC heme iron oxidant. Dimethylsulfoxide (DMSO) vehicle or varying concentrations of GSK2181236A (2 pM to 1 μM) or BAY 60-4552 (15 nM to 100 μM) were then added. Following a 30 min incubation at 37°C, media was aspirated and the cells were rinsed with phosphate-buffered saline (PBS) and fixed with 4% formaldehyde in PBS by incubating at room temperature for 20 min. Cells were then washed with PBS and permeabilized for 10 min using 0.1% triton X-100 in PBS. Following PBS rinsing, the cells were blocked for 90 min at room temperature with Odyssey Blocking Buffer (LI-COR, Cambridge, UK). The buffer was aspirated and the cells were treated overnight at 4°C with primary antibody [(pSer^239^) VASP, rabbit polyclonal Ab, Cell Signaling Technology, Danvers, MA, USA] diluted 1:500 in Odyssey Blocking Buffer. Following three washes with 0.05% Tween 20, the cells were treated for 1 h at room temperature with secondary antibody (IRDye^®^ 800CW Donkey Anti-Rabbit IgG, LI-COR) diluted 1:2500 in Odyssey Blocking Buffer with 0.05% Tween 20. Following two washes with PBS, infrared fluorescence was measured using an Odyssey Infrared Imaging System (LI-COR).

### *In vitro* selectivity profile of GSK2181236A

The secondary pharmacological profile (i.e., sGC-independent activity) of GSK2181236A was assessed in cell-based functional assays by characterizing the interaction between GSK2181236A and 38 distinct human enzymes, ion channels, transporters, and G-protein-coupled receptors including 5-HT (5-HT_1B/2A/2C/3_), 5-HT transporter (SERT), adenosine (A_2a_), adrenergic (α_1B/2C_, β_2_), Aurora B kinase, cycoloxygenase-2 (COX-2), Ca_V_1.2, cytochrome P450 (CYP2C19, 2D6, and 3A4), dopamine (D_2_), dopamine transporter (DAT), gamma-aminobutyric acid (GABA_A_), glycogen synthase kinase-3 (GSK3β) histamine (H_1_), K_V_1.5, K_V_7.1, K_V_11.1, lymphocyte-specific protein tyrosine kinase (LCK), l-monoamine oxidase (MAO-B) muscarinic (M_1/2_), Na_V_1.5, nicotinic (α1 nAChR), noradrenaline transporter (NET), opioid (κ, μ), organic anion transporting polypeptide C (OATP1B1), phosphodiesterase 5 (PDE_5_), phosphatidylinositol 3-kinase (PI3Kγ), pregnane X receptor (PXR), tachykinin (NK_1_), and vasopressin (V_1a_).

### Hemodynamic assessment of GSK2181236A and BAY 60-4552 in sprague dawley rats

To identify equi-efficacious doses of GSK2181236A and BAY 60-4552, heart rate and blood pressure were monitored in male Sprague Dawley (SD) rats (∼500g, *n* = 4–6 per group) via surgically implanted radiotelemetry transmitters (PhysioTel PA-C40, Data Sciences International, St. Paul, MN, USA). Following oral gavage dosing (0.03–3.0 mg/kg), hemodynamic data were recorded every 5 min for 10 s and then averaged every hour. Since both compounds demonstrated transient effects on heart rate and blood pressure, data are presented as an average change from vehicle over an 11 h period post-dose.

### Myocardial I/R injury and hemodynamic assessment of GSK2181236A and BAY 60-4552 in SD rats

This study was performed at CV Dynamics, Inc. (Newark, NJ, USA). Male SD rats were obtained from Charles River Laboratories, Inc. (Kingston, NY, USA). At 2 months of age, rats were pre-medicated with atropine (0.04 mg/kg IM) then anesthetized with a loading dose of pentobarbital sodium (65 mg/kg IP). A supplemental dose of pentobarbital sodium (16.3 mg/kg IP) was administered 30–40 min after the initial anesthesia. Marcaine (0.025–0.05 mg/rat IM) was administered locally for pain control, and anesthetized rats were subjected to a thoracotomy at the fourth intercostal space. The left descending coronary artery was occluded using a 5-0 silk snare for 30 min, followed by reperfusion for 24 h. The coronary occlusion and reperfusion were verified by visual inspection (color change of myocardium from pale to pink) and by EKG monitoring [ST elevation and widening of the QRS; Gould BioTACH ECG module (Valley View, OH, USA) with a Tektronix TDS 220 monitor (Beaverton, OR, USA)]. A 2% lidocaine solution (2–3 mg/kg) was dripped directly onto the heart as needed for arrhythmia control during ischemia, and procainamide (12.5 mg/kg IP) was administered 15 min prior to reperfusion.

Rats were orally gavaged with vehicle (0.5% HPMC, 5% DMSO, and 0.1% Tween 80; 10 mL/kg; *n* = 14), GSK2181236A (0.1 or 1.0 mg/kg; *n* = 11–14), or BAY 60-4552 (0.3 or 3.0 mg/kg; *n* = 10–12) 2 h prior to ischemia. Blood was collected at the initiation of ischemia and after 24 h reperfusion. Plasma was obtained for analysis of compound concentrations and levels of cardiac Troponin I (cTnI), a circulating biomarker of cardiac injury. Plasma concentrations of cTnI were determined using a commercially available ELISA (catalog #2010-2-HSP, Life Diagnostics, West Chester, PA, USA) according to the manufacturer’s instructions. Plasma compound concentrations were determined by LC/MS/MS (API 5000 triple quadrupole mass spectrometer, ABSciex, Foster City, CA, USA; Prominence liquid chromatographic system, Shimadzu, Columbia, MD, USA). Rats were anesthetized with a mixture of ketamine and xylazine (35 and 5 mg/kg, IM) and euthanized via exsanguination. Hearts were excised and perfused with 1% Evans blue dye in saline then perfused-fixed with 10% neutral buffered formalin (∼90 mmHg perfusion pressure). Hearts were sliced on the short axis from apex to base at 1–2 mm intervals, and the ischemic area (area at risk) was evaluated using Image Pro-Plus (Media Cybernetics, Inc., Bethesda, MD, USA). Fresh heart slices were stained with 1% triphenyltetrazolium and evaluated using Image Pro-Plus for determination of infarct size.

### Cardiac and renal function assessment of GSK2181236A and BAY 60-4552 in HSFD fed SHR-SP

The SHR-SP is an established model of genetic hypertension. Exposure to a HSFD exacerbates endothelial dysfunction, systemic vascular resistance and hypertension, and accelerates end-organ damage (Ma et al., 2001). Decreases in endothelium-dependent vasorelaxation in this model have been associated with reduced levels of NO bioactivity and increased generation of superoxide and/or peroxynitrite anions (McIntyre et al., 1997, 1999; Kerr et al., 1999; Ma et al., 2001; see Bauersachs and Widder, 2008 for review). As such, a cohort of HSFD SHR-SP was established as a model of cardiovascular disease associated with chronic oxidative stress in this study.

Male SHR-SPs (*n* = 75) were obtained from Charles River Laboratories, Inc. (Kingston, NY, USA) and maintained on a normal powdered rodent diet (Lab Diet 5001, PMI Nutrition International, LLC, St. Louis, MO, USA) and tap water *ad libitum*. A subgroup of animals (*n* = 45) was anesthetized with isoflurane and surgically implanted with radiotelemetry transmitters (PhysioTel PA-C40, Data Sciences International, St. Paul, MN, USA). The transmitter catheter was threaded through the femoral artery into the abdominal aorta. At 11–12 weeks of age, baseline blood pressure and heart rate were measured for 5 days (10 s recordings every 5 min) in normal caging, then rats were acclimated to metabolic caging for a minimum of 48 h before a basal 24 h urine sample and blood were collected. After baseline measures, rats were assigned to six groups (*n* = 8–14 per group) according to mean arterial pressure (MAP; telemetered rats only) and body weight: normal diet (ND) control, HSFD, HSFD + 0.1 mg/kg/day GSK2181236A, HSFD + 1.0 mg/kg/day GSK2181236A, HSFD + 0.3 mg/kg/day BAY 60-4552, and HSFD + 3.0 mg/kg/day BAY 60-4552. The ND group was maintained on normal chow and water throughout the study. The other groups received a fat-enriched rodent diet (24.5% fat, Zeigler Bros., Inc., Gardners, PA, USA) and a 1% NaCl drinking water solution *ad libitum* for 8 week (Willette et al., 2009). Rats displaying signs of morbidity which included piloerection, lack of grooming, persistent body weight loss, lethargy, or seizure/convulsions, were promptly euthanized.

Treatments were administered in respective diets starting 1 week after the initiation of HSFD. Urine collections were repeated on weeks 2, 4, 6, and 8 and blood was sampled in the morning after each 24 h collection period for assessment of renal function. Plasma creatinine and urine sodium, microalbumin (MALB), and creatinine were ascertained using an AU640 Chemistry Analyzer (Olympus America, Inc., Center Valley, PA, USA). Plasma compound concentrations were determined as described above.

During week 7, cardiac function and morphometry were evaluated via transthoracic echocardiography. Rats were anesthetized with isoflurane (2–4% induction and 1–3% maintenance). Two-dimensional gray scale images of the left ventricle were obtained in the short axis plane at mid-ventricle level and in the parasternal long axis plane using a GE Vivid 7 system (GE Medical Systems, Milwaukee, WI, USA) and stored as digital cineloops. Pulse wave Doppler recordings from the LV outflow were obtained to determine heart rate. All images were analyzed by a single observer using a GE EchoPac (GE Medical Systems, Milwaukee, WI, USA). LV volumes were calculated as $V=\frac{5}{6}(\text{LVA}\times \text{LVL)}$ where LVA is area of LV chamber from the short axis view and LVL is the LV length from the long axis view at end diastole and end systole. LV mass was calculated from two dimensional images using the area-length method (Lang et al., 2005). Analysis was performed on three to five cardiac cycles.

On week 8, isoflurane anesthetized rats were euthanized via exsanguination. The thoracic aorta was excised for assessment of endothelial function as described below. Hearts were snap-frozen in liquid nitrogen and stored at −80°C for later analysis of gene expression changes associated with cardiac hypertrophy and fibrosis (see below for procedure details).

### Gene expression analysis of cardiac tissue

Cardiac gene expression was analyzed following chronic (7 week) BAY 60-4552 or GSK2181236A treatment in HSFD SHR-SP. Hearts were homogenized in Trizol and RNA was extracted using the RNeasy Plus Mini kit protocol (Qiagen, Valencia, CA, USA) with a 40 μL elution volume. RNA was quantified using a Nanodrop Spectrophotometer ND-1000 (Thermo Fisher Scientific, Wilmington, DE, USA) and converted to cDNA using the High Capacity cDNA Reverse Transcription Kit (Applied Biosystems, Carlsbad, CA, USA). Taqman assays were performed using a 384-well plate format with individual primer-probes and Taqman Universal PCR Master Mix (Applied Biosystems) and run on the 7900 HT Fast Real-Time PCR System (Applied Biosystems). SDS 2.3 software (Applied Biosystems) was used to obtain raw *C*_t_ values with manual threshold set at 0.10. Array Studio software (version 3.6.2; OmicSoft Corporation, Cary, NC, USA) was used to normalize gene expression data, by analysis of covariance, to the housekeeper genes β-2-microglobulin (*B2m*) and peptidyl-prolyl cis-trans isomerase A (*Ppia*). No outliers were detected by means of principal component analysis.

The following genes were analyzed: α-skeletal actin (*Acta1*), type 1 collagen-alpha 1 (*Col1a1*), type 3 collagen-alpha 1 (*Col3a1*), connective tissue growth factor (CTGF; *Ctgf*), matrix metalloproteinase 2 (MMP2; *Mmp2*), matrix metalloproteinase 9 (MMP9; *Mmp9*), α-myosin heavy chain (α-MHC; *Myh6*), β-myosin heavy chain (β-MHC; *Myh7*); atrial natriuretic factor (ANF; *Nppa*), brain-type natriuretic peptide (BNP; *Nppb*), cGMP-specific phosphodiesterase 5A (PDE5A; *Pde5a*), type 2 cGMP-dependent protein kinase (PKG2; *Prkg2*), and transforming growth factor beta 1 (TGF-β1; *Tgfb1*).

### Isolated artery vasodilatation

Male Wistar Kyoto (WKY; Charles River Laboratories, Inc., Raleigh, NC, USA) were anesthetized with inhaled isoflurane (5% in O_2_) and euthanized by exsanguination. Thoracic aorta was isolated, cleaned of adherent tissue, cut into 3 mm rings, and suspended in 10 mL organ baths containing Krebs–Henseleit buffer (KHB) of the following composition (mM): NaCl, 112.0; KCl, 4.7; KH_2_PO_4_, 1.2; MgSO_4_, 1.2; CaCl_2_, 2.5; NaHCO_3_, 25.0; glucose, 11.0. KHB was maintained at 37°C and aerated with 95%O_2_:5%CO_2_ (pH 7.4). Changes in isometric force were measured under 1g optimal resting tension using MLT0201/D force-displacement transducers (Letica, Barcelona, Spain) and recorded digitally (Chart 5.0 software, ADInstruments, Colorado Springs, CO, USA). After a 1 h equilibration period, each tissue was contracted to equilibrium with 60 mM KCl, washed with KHB and allowed to relax to the resting tension. The 60 mM KCl contraction was repeated and subsequent contractile responses were normalized to this second KCl response. Each tissue was then contracted to equilibration with 1 μM phenylephrine, washed with KHB and allowed to relax to the resting tension. In order to further investigate the mechanism by which BAY 60-4552 and GSK2181236A were modulating sGC function, the vasodilatory effects of both compounds were assessed in the presence and absence of ODQ, a heme-site inhibitor of sGC. Following a 20 min pre-treatment with DMSO vehicle (0.1% final concentration, v/v) or ODQ (10 μM), aortae were contracted with 0.3 μM phenylephrine (an approximate EC_80_ concentration). Once the contractile response to phenylephrine reached a plateau (∼10 min), contractile tone was reversed by adding cumulative concentrations of BAY 60-4552 or GSK2181236A to the tissue baths at log increments (0.1 nM to 10 μM).

In separate studies, endothelial function was assessed following chronic (7 week) BAY 60-4552 or GSK2181236A treatment in SHR-SP on HSFD. SHR-SP fed either normal or HSFD served as controls. Rats were anesthetized and thoracic aorta were isolated and treated with KCl and phenylephrine as described above. Cumulative concentration-response curves to phenylephrine were obtained by adding 0.5 log unit increments (1 nM to 3 μM). Following several washes with KHB, each vessel was contracted to equilibrium with 0.3 μM phenylephrine and tone was reversed by adding cumulative amounts of carbachol at 0.5 log unit intervals (10 nM to 30 μM). Following several KHB washes, the tissues were again contracted to equilibrium with 0.3 μM phenylephrine and tone was reversed by adding cumulative amounts of SNP at 0.5 log unit intervals (0.1 nM to 3 μM). In separate tissues from both the normal and HSFD SHR-SP control groups, the direct vasodilatory effects of BAY 60-4552 and GSK2181236A were investigated. Contractile tone induced by 0.3 μM phenylephrine was reversed by adding cumulative concentrations of BAY 60-4552 or GSK2181236A to the tissue baths at log increments (0.1 nM to 10 μM).

### Materials

BAY 60-4552 and GSK2181236A were synthesized by GlaxoSmithKline (King of Prussia, PA, USA). DMSO and ODQ were from EMD Chemicals (Gibbstown, NJ, USA), phenylephrine, carbachol, SNP, HPMC, Tween 80, Evan’s blue dye, and triphenyltetrazolium dye were from Sigma (St Louis, MO, USA) and isoflurane was from Abbott Laboratories (North Chicago, IL, USA). All medications administered to animals during survival and non-survival procedures were of pharmaceutical grade. All other reagents were of analytical grade.

### Data analysis

Data are presented as mean ± SEM unless indicated otherwise. For analysis of physiologic and contractility data, statistical comparisons were made using two-tailed *t*-tests, or one- or two-way ANOVA analysis with a Dunnett’s or Bonferroni’s post-test, respectively, where *P < 0.05, **P < 0.01, and ***P < 0.001. For gene expression analysis, fold changes and statistical significance were determined by performing one-way ANOVA with False Discovery Rate – Benjamini and Hochberg (FDR-BH) multiplicity where *P < 0.05, **P < 0.01, and ***P < 0.001. Concentration-dependent P-VASP formation, vasoconstriction and vasodilation curves were fit by non-linear regression (Graphpad Prism 5.0, La Jolla, CA, USA). In cases where non-linear regression analysis could not be accurately performed due to the lack of complete concentration-response curves (e.g., vasodilatory responses in the presence of ODQ), individual responses to each concentration of vasodilator were compared. Survival curves were generated using the Kaplan–Meier method and each curve was individually compared to HSFD control SHR-SP using the Mantel–Cox log-rank test (GraphPad Prism 5.0) where statistical difference was designated as *P* < 0.05.

## Results

### Effects of ODQ on GSK2181236A- and BAY 60-4552-mediated P-VASP formation and vasodilation

**Figure 2:**
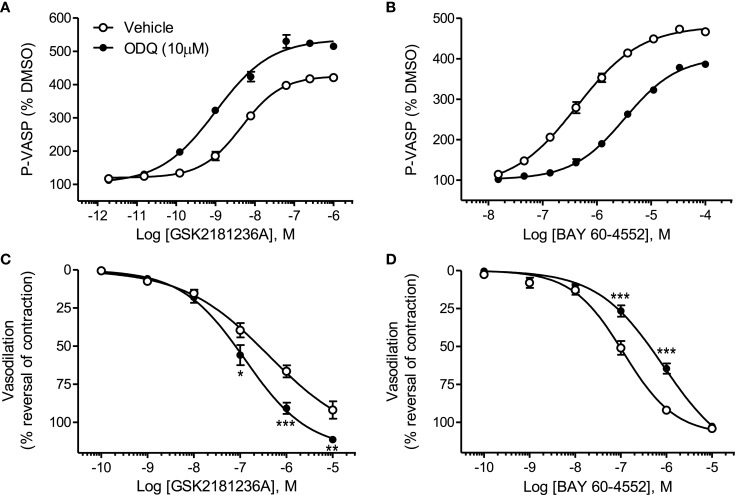
**Effects of the sGC heme-site inhibitor ODQ on GSK2181236A- and BAY 60-4552-mediated P-VASP formation and vasodilation**. **(A)** GSK2181236A increased P-VASP levels in a concentration-dependent manner in rat aortic smooth muscle cells and these effects were potentiated by pre-treatment with 10 μM ODQ. **(B)** BAY 60-4552 increased P-VASP formation, but, in contrast to GSK2181236A, the concentration-dependent effects of BAY 60-4552 were attenuated by ODQ. P-VASP data are representative from one experiment performed in triplicate and are expressed as mean ± SEM (consolidated pEC_50_ and *E*_max_ values and statistical comparisons are provided in Table 1). Consistent with the P-VASP assay results, ODQ **(C)** augmented the vasodilatory effects of the sGC activator GSK2181236A in WKY rat isolated aorta and **(D)** attenuated the vasodilatory effects of BAY 60-4552. Vasodilatory data are expressed as mean ± SEM of 6–10 animals. Non-linear regression analysis of several vasodilation concentration-response curves in the presence of ODQ could not be accurately performed due to the lack of a complete sigmoidal response; therefore, statistical comparisons of vehicle vs. ODQ were made at each concentration of vasodilator using two-way ANOVA analysis with a Bonferroni’s post-test where *P < 0.05, **P < 0.01, and ***P < 0.001.

GSK2181236A increased P-VASP levels in a concentration-dependent manner with an EC_50_ of 12.7 nM (Figure 2A; Table 1). This value is consistent with primary cell-free rat and human sGC enzyme assays where GSK2181236A increased sGC activity (cGMP formation) with EC_50_s of 27 and 25 nM, respectively (not shown). Pre-treatment with the heme-site sGC inhibitor ODQ (10 μM) potentiated the effects of GSK2181236A, causing a 4.7-fold leftward-shift to the concentration-response curve and augmenting the P-VASP maximal response by 32% (Figure 2A; Table 1). The NO-sensitive sGC stimulator BAY 60-4552 also increased P-VASP levels in a concentration-dependent manner. However, in contrast to GSK2181236A, ODQ attenuated BAY 60-4552-induced P-VASP formation, causing a 9.8-fold rightward-shift to the BAY 60-4552 concentration-response curve and a trend toward suppressing the maximal response by 7% (Figure 2B; Table 1).

**Table 1:** **Effects of GSK2181236A or BAY 60-4552 on P-VASP formation in rat aortic smooth muscle cells**.

	EC_50_ (nM)		*E*_max_ (% DMSO)	
	Control	ODQ (10 μM)	Control	ODQ (10 μM)
GSK2181236A	12.7 ± 4.7	2.7 ± 0.6**	379 ± 26	502 ± 26*
BAY 60-4552	353 ± 89	3,264 ± 737***	388 ± 39	362 ± 24

All values are expressed as mean ± SEM (*n* = 4 independent experiments performed in at least duplicate). *E*_max_ values are expressed as relative fluorescence units normalized to DMSO vehicle. Statistical comparisons of vehicle- and ODQ-treated cells were performed using two-tailed *t*-tests where *P < 0.05, **P < 0.01, and ***P < 0.001 vs. vehicle control.

Consistent with the P-VASP assay results, ODQ augmented the vasodilatory effects of the sGC activator GSK2181236A. Specifically, ODQ increased the vasodilatory responses to 0.1, 1, and 10 μM GSK2181236A by 41, 36, and 21%, respectively (Figure 2C). In contrast to GSK2181236A, the vasodilatory response to the sGC stimulator BAY 60-4552 was attenuated by ODQ treatment (ODQ reduced responses to 0.1 and 1 μM BAY 60-4552 by 48 and 30%, respectively; Figure 2D). These results confirm the mechanism of action proposed for BAY 60-4552 (NO-sensitive sGC stimulation; Mitrovic et al., 2009) and indicate that GSK2181236A increases sGC enzymatic activity in a NO-insensitive manner. In addition, these results support the hypothesis that activation of NO-insensitive sGC, but not stimulation of NO-sensitive sGC, will be potentiated under conditions of oxidative stress.

### *In vitro* selectivity profile of GSK2181236A

The most potent interaction between GSK2181236A and a “non-sGC” protein occurred with OATP1B1, where the compound inhibited the transporter activity of this protein with an IC_50_ of 0.3 μM. Interactions (EC_50_s or IC_50_s) of ≥2.5 μM were observed at all other proteins tested. As such, considering GSK2181236A activates sGC with an EC_50_ of 12.7 nM (P-VASP formation; Table 1), GSK2181236A functioned as a selective sGC activator with 23-fold selectivity for sGC over OATP1B1 and ≥197-fold selectivity for all other tested proteins.

### Identification of equi-efficacious doses of GSK2181236A and BAY 60-4552 in SD rats

**Figure 3:**
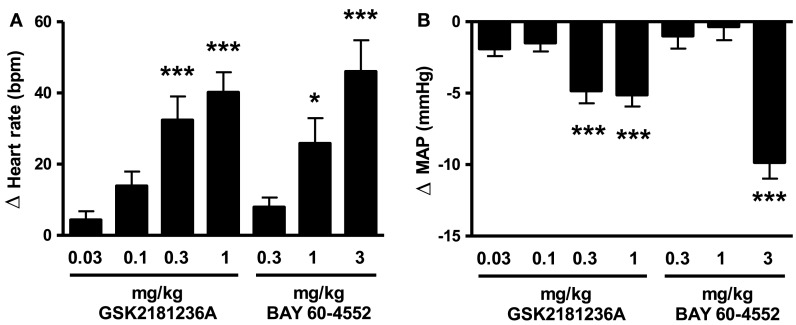
**Effect of GSK2181236A and BAY 60-4552 on (A) heart rate and (B) mean arterial pressure (MAP) in normotensive Sprague Dawley rats**. A single p.o. dose of compounds in radio telemetered rats resulted in a dose-dependent change in heart rate and blood pressure. Data are presented as an average change from vehicle over an 11 h period post-dose. All data are mean ± SEM of four to six animals. Statistical comparisons to vehicle were performed for each dose using one-way ANOVA with a Dunnett’s post-test where *P < 0.05, **P < 0.01, and ***P < 0.001.

The sGC activator GSK2181236A increased heart rate and reduced blood pressure at doses ≥0.3 mg/kg (Figure 3). The sGC stimulator BAY 60-4552 was approximately threefold less potent than GSK2181236A (Figure 3). Doses for the I/R and HSFD SHR-SP studies were selected based upon the highest no-effect dose (0.1 mg/kg GSK2181236A and 0.3 mg/kg BAY 60-4552) and a dose 10-fold higher which significantly changed heart rate and mean blood pressure (1 mg/kg GSK2181236A and 3 mg/kg BAY 60-4552; Figure 3).

### Effects of GSK2181236A and BAY 60-4552 on I/R injury in SD rats

**Figure 4:**
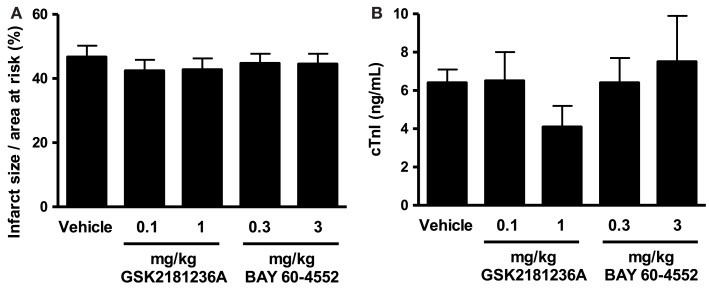
**Effect of GSK2181236A and BAY 60-4552 on cardiac ischemia/reperfusion injury in Sprague Dawley rats (*n* = 10–14 per group)**. Acute p.o. administration of compounds did not significantly alter **(A)** infarct size as a percentage of the area at risk or **(B)** circulating levels of cardiac troponin I (cTnI), an indicator of cardiac injury. Data are presented as mean ± SEM. Statistical comparisons to vehicle were performed using one-way ANOVA with a Dunnett’s post-test where significance was determined as *P* < 0.05.

Cardiac I/R resulted in an infarct size/area at risk of 46.7 ± 3.5% (Figure 4A) and plasma cTnI levels of 6.4 ± 0.7 ng/mL in vehicle-treated rats (Figure 4B). Neither GSK2181236A (0.1 and 1.0 mg/kg) nor BAY 60-4552 (0.3 and 3 mg/kg) significantly altered either endpoint (Figure 4), suggesting that activation of NO-insensitive sGC does not provide an advantage over stimulation of NO-sensitive sGC with regard to protecting the myocardium from ischemic injury. Plasma concentrations of GSK2181236A and BAY 60-4552 were consistent with levels observed in the SD hemodynamic study (data not shown). Mortality rates were similar between all groups: sham, 0 of 4; vehicle, 1 of 15 (due to anesthesia); 0.1 mg/kg GSK2181236A, 1 of 15 (due to anesthesia), 1 mg/kg GSK2181236A, 2 of 13 (due to arrhythmia during occlusion and unknown cause during reperfusion); 0.3 mg/kg BAY 60-4552, 2 of 12 (due to arrhythmia and trauma during occlusion); 3 mg/kg BAY 60-4552, 2 of 14 (due to cardiac puncture and trauma).

### Effects of GSK2181236A and BAY 60-4552 on blood pressure, renal and cardiac function, and survival in SHR-SP

Chronic dietary administration of GSK2181236A and BAY 60-4552 sustained dose-linear plasma concentrations that were consistent with levels observed previously in the SD hemodynamic and I/R studies (data not shown).

**Figure 5:**
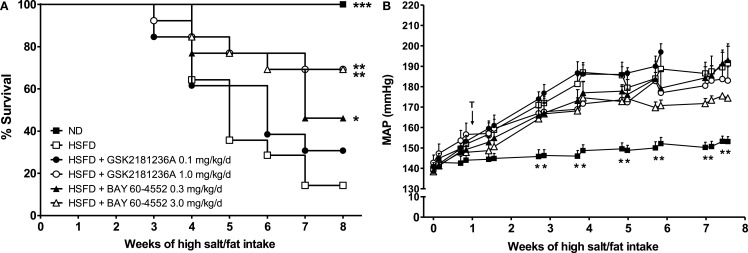
**Chronic administration of GSK2181236A and BAY 60-4552 enhanced (A) survival and similarly, but not significantly, reduced (B) blood pressure in age-matched, radiotelemetered HSFD SHR-SPs**. Treatments (T) were administered in chow starting 1 week after the initiation of HSFD. Average daily blood pressure measurements (10 s recordings measured every 5 min for 24 h) are reported for 2 days/week for the duration of the study. Blood pressure data were analyzed using two-way ANOVA analysis with a Bonferroni’s post-test and statistical comparisons were made to HSFD control (**P < 0.01; data are presented as mean ± SEM of two to eight animals per group per time point). Survival curves were generated using the Kaplan–Meier method. Each curve was individually compared to HSFD SHR-SP using the Mantel–Cox log-rank test where ***P < 0.001, **P < 0.01, and *P < 0.05.

Whereas the survival rate of the ND SHR-SPs was 100%, 8 week of HSFD treatment reduced survival to only 14% (*P* < 0.001 vs. ND; Figure 5A). Treatment for 7 week with 0.1 mg/kg/day GSK2181236A did not improve survival; however, the higher dose (1.0 mg/kg/day) increased the survival rate to 69% (*P* < 0.01 vs. HSFD; Figure 5A). In contrast, both the 0.3 and 3 mg/kg/day doses of BAY 60-4552 improved survival (46 and 69%, *P* < 0.01, and *P* < 0.05 vs. HSFD, respectively; Figure 5A).

In the ND group, MAP averaged 142 ± 4 mmHg at the start of study and increased to 153 ± 2 mmHg after 8 week (Figure 5B). In contrast to ND, the MAP in SHR-SPs fed a HSFD for 8 week increased from 140 ± 2 to 192 ± 10 mmHg (*P* > 0.05 and *P* < 0.01 vs. ND, respectively; Figure 5B). Compared to the HSFD controls, treatment for 7 week with GSK2181236A (1.0 mg/kg/day) mildly, but not significantly, reduced MAP by 9 to 183 ± 7 mmHg (*P* > 0.05; Figure 5B). Seven weeks of BAY 60-5442 (3 mg/kg/day) reduced MAP to a greater extent than 1.0 mg/kg/day GSK2181236A, reducing MAP by 17 to 175 ± 1 mmHg (*P* > 0.05; Figure 5B). The lower doses of both compounds (0.1 mg/kg/day GSK2181236A and 0.3 mg/kg/day BAY 60-4552) did not have any effect on MAP compared to the HSFD controls (Figure 5B).

**Figure 6:**
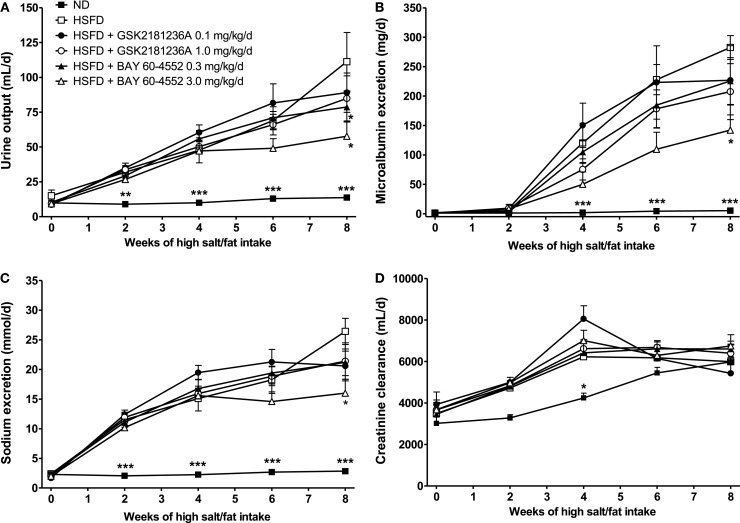
**Chronic administration of BAY 60-4552, but not GSK2181236A attenuated the increase in (A) urine output, (B) microalbumin excretion, and (C) sodium excretion in age-matched HSFD SHR-SPs**. Neither compound altered glomerular filtration rate as estimated by **(D)** creatinine clearance. Treatments were administered in chow starting 1 week after the initiation of HSFD. Data for moribund animals or animals that died prior to the end of study were not included. Remaining data were analyzed using two-way ANOVA analysis with a Bonferroni’s post-test and statistical comparisons were made to the HSFD control (*P < 0.05 and ***P < 0.001; data are presented as mean ± SEM of three to nine animals per group).

Urine output in the ND group was 10 ± 1 mL/day under basal conditions and 14 ± 1 mL/day at 8 week (*P* < 0.01 vs. baseline; Figure 6A). Similarly, MALB excretion was 1.3 ± 0.2 mg/day at baseline and 5.3 ± 0.8 mg/day after 8 week (*P* < 0.001 vs. baseline; Figure 6B), and sodium excretion was 2.3 ± 0.2 and 2.8 ± 0.1 mmol/day at baseline and 8 week, respectively (*P* < 0.05 vs. baseline; Figure 6C). Eight weeks of HSFD increased urine output in SHR-SPs from 15 ± 4 to 111 ± 21 mL/day (*P* > 0.05 and *P* < 0.001 vs. ND, respectively; Figure 6A), increased MALB excretion from 1.3 ± 0.03 to 282.4 ± 20.2 mg/day (*P* > 0.05 and *P* < 0.001 vs. ND, respectively; Figure 6B), and increased sodium excretion from 2.4 ± 0.4 to 26.4 ± 2.2 mmol/day (*P* > 0.05 and *P* < 0.001 vs. ND, respectively; Figure 6C). Compared to the HSFD controls, GSK2181236A (0.1 and 1.0 mg/kg/day) did not alter urine output, MALB excretion, or sodium excretion (Figures 6A–C). Seven weeks of treatment with BAY 60-4552 (0.3 and 3.0 mg/kg/day) dose-dependently decreased urine output to 79 ± 11 and 56 ± 10 mL/day (*P* < 0.05 vs. HSFD; Figure 6A). BAY 60-4552 (3.0 mg/kg/day) also reduced MALB excretion to 142 ± 42 mg/day and sodium excretion to 16 ± 2 mmol/day (*P* < 0.05 vs. ND; Figures 6B,C). Glomerular filtration rates estimated by creatinine clearance were similar in ND and HSFD SHR-SPs at baseline and at the end of study (*P* > 0.05 vs. ND), and were not altered by treatment with GSK2181236A or BAY 60-4552 (*P* > 0.05 vs. HSFD; Figure 6D). Overall, these data suggest that stimulation of NO-sensitive sGC delays development of renal dysfunction more effectively than activation of NO-insensitive sGC. However, it should be noted that the renal protection afforded by BAY 60-4552 might be a direct result of blood pressure modulation (BAY 60-4552 reduced blood pressure to a greater extent than GSK2181236A; see above).

Upon echocardiographic analysis, as compared to ND, HSFD produced mild LV hypertrophy [LV mass to body weight ratio (LVM/BW) was increased by 15%; *P* < 0.05] with preserved ejection fraction (Table 2). LVM/BW was reduced by treatment with 0.1 and 1.0 mg/kg/day GSK2181236A to ND levels (*P* < 0.05 vs. HSFD; Table 2). BAY 60-4552 (3.0 mg/kg/day) similarly, but not significantly, reduced LVM/BW to near ND levels (*P* > 0.05; Table 2). No other morphometric or functional parameters were altered by GSK2181236A or BAY 60-4552 as compared to HSFD (Table 2). Thus, although both compounds attenuated the development of cardiac hypertrophy, only activation of sGC by GSK2181236A did so in a manner independent of changes in blood pressure.

**Table 2:** **Effects of chronic GSK2181236A or BAY 60-4552 treatment on echocardiographic parameters in SHR-SP**.

			HSFD + GSK2181236A		HSFD + BAY 60-4552	
	ND (*n* = 8)	HSFD (*n* = 11)	0.1 mg/kg/day (*n* = 6)	1.0 mg/kg/day (*n* = 10)	0.3 mg/kg/day (*n* = 9)	3.0 mg/kg/day (*n* = 10)
BW (g)	332 ± 3	315 ± 7	331 ± 16	338 ± 5	328 ± 6	345 ± 14
EDV (μL)	39 ± 16	330 ± 15	331 ± 29	332 ± 22	356 ± 21	379 ± 21
ESV (μL)	141 ± 10*	102 ± 7	110 ± 11	98 ± 10	111 ± 11	120 ± 9
EF (%)	65 ± 1	69 ± 1	67 ± 2	71 ± 1	69 ± 2	67 ± 1
SV (μL)	255 ± 9	229 ± 10	222 ± 19	234 ± 14	245 ± 14	259 ± 13
CO (mL/min)	89 ± 4	80 ± 4	76 ± 7	80 ± 5	86 ± 6	89 ± 6
LVM/BW (mg/g)	2.68 ± 0.03*	3.08 ± 0.06	2.69 ± 0.08*	2.82 ± 0.07*	3.06 ± 0.07	2.85 ± 0.10

Echocardiographic analysis was performed after 7 weeks of HSFD. Treatments were administered in chow starting 1 week after initiation of HSFD. All values are expressed as mean ± SEM. ND, normal diet; HSFD, high salt/fat diet; BW, body weight at week 7; EDV, end diastolic volume; ESV, end systolic volume; EF, ejection fraction; SV, stroke volume; CO, cardiac output; LVM/BW, left ventricular mass to body weight ratio. Statistical comparisons were performed using one-way ANOVA analysis with a Dunnett’s post-test where *P < 0.05 vs. HSFD control SHR-SP.

### Effects of GSK2181236A and BAY 60-4552 on cardiac gene expression in SHR-SP

**Figure 7:**
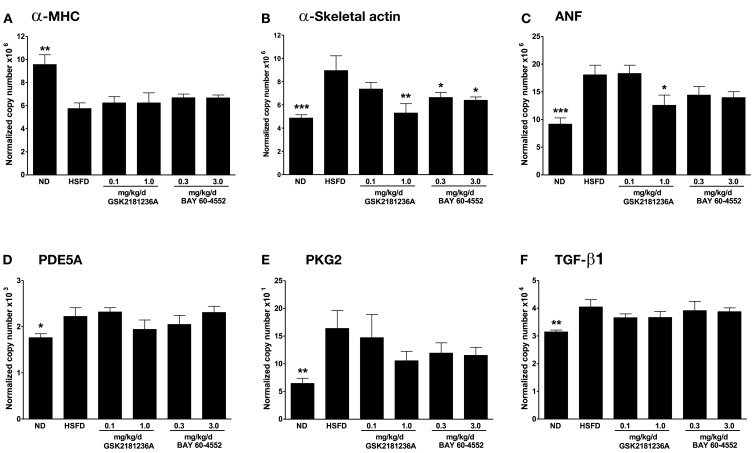
**Effect of GSK2181236A and BAY 60-4552 on cardiac mRNA expression following 8 weeks of HSFD in SHR-SPs**. HSFD reduced the myocardial expression of **(A)** α-myosin heavy chain (α-MHC; *Myh6*), an indicator of reduced cardiac contractility, and increased the expression of the pathological hypertrophy and fibrosis gene markers **(B)** α-skeletal actin (*Acta1*), **(C)** atrial natriuretic factor (ANF; *Nppa*), **(D)** cGMP-specific phosphodiesterase 5A (PDE5A; *Pde5a*), **(E)** type 2 cGMP-dependent protein kinase (PKG2; *Prkg2*), and **(F)** transforming growth factor beta 1 (TGF-β1; *Tgfb1*). Treatments were begun 1 week after initiation of HSFD and attenuated the HSFD-induced increases of **(B)** α-skeletal actin and **(C)** ANF expression to near normal diet (ND) levels. Gene expression data were normalized to levels of transcript encoding β-2-microglobulin (*B2m*) and peptidyl-prolyl cis-trans isomerase A (*Ppia*). All data are mean ± SEM of four to nine animals. Statistical comparisons were performed using one-way ANOVA, with False Discovery Rate – Benjamini and Hochberg (FDR-BH) multiplicity, comparing respective groups to the HSFD control group (*P < 0.05, **P < 0.01, and ***P < 0.001).

Compared to ND SHR-SP, HSFD reduced the cardiac expression of α-MHC by 1.6-fold (*P* < 0.01; Figure 7A) and increased expression of α-skeletal actin by 1.8-fold (*P* < 0.001; Figure 7B), ANF by 2.0-fold (*P* < 0.001; Figure 7C), PDE5A by 1.3-fold (*P* < 0.05; Figure 7D), PKG2 by 2.6-fold (*P* < 0.01; Figure 7E), and TGF-β1 by 1.3-fold (*P* < 0.01; Figure 7F). Eight weeks of HSFD did not alter cardiac expression of BNP, CTGF, type 1 collagen-alpha 1, type 3 collagen-alpha 1, MMP2, or MMP 9 (data not shown).

After 7 weeks of treatment, 1.0 mg/kg/day GSK2181236A attenuated the HSFD-induced increase of α-skeletal actin and ANF by 1.7- and 1.4-fold, respectively (*P* < 0.01 and *P* < 0.05 vs. HSFD, respectively; Figures 7B,C).

Similarly, 0.3 and 3.0 mg/kg/day BAY 60-4552 attenuated the HSFD-induced increase of α-skeletal actin by 1.3- and 1.4-fold, respectively (*P* < 0.05 vs. HSFD; Figure 7B), but only exhibited a non-significant trend toward attenuating the increase of ANF (*P* > 0.05 vs. HSFD; Figure 7C). Expression of α-MHC, PDE5A, PKG2, and TGF-β1 was not altered by treatment with GSK2181236A or BAY 60-4552 (Figures 7A,D–F). Overall, GSK2181236A and BAY 60-4552 exhibited similar cardioprotective gene expression profiles.

### Vasodilatory effects of carbachol, SNP, BAY 60-4552, and GSK2181236A in SHR-SP

**Figure 8:**
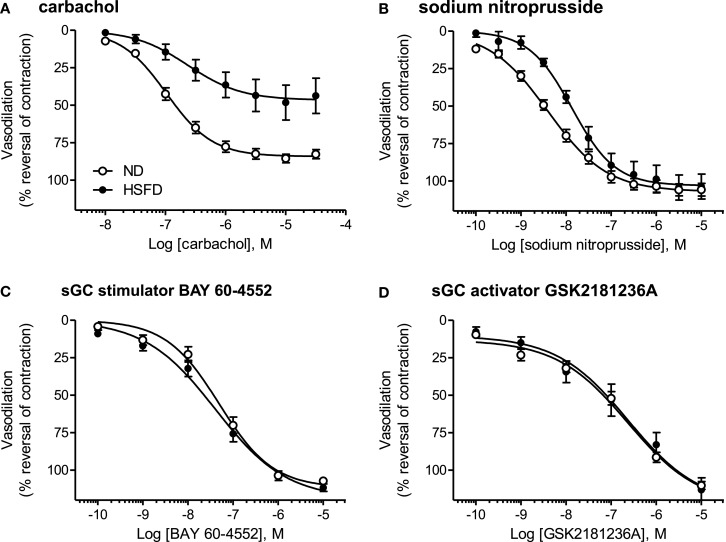
**Direct vasodilatory effects of various NO/sGC/cGMP pathway modulators in aorta isolated from ND and HSFD SHR-SP**. **(A)** As compared to ND SHR-SP, the maximal vasodilatory response to carbachol, an endothelium- and NO-dependent vasodilator, was suppressed in HSFD SHR-SP. **(B)** The vasodilatory response to the endothelium-independent vasodilator and NO-donor SNP was also inhibited in HSFD SHR-SP, being right-shifted 3.5-fold as compared to the response observed in ND animals. In contrast to the blunted vasodilatory responses seen with the NO-dependent vasodilators carbachol and SNP, chronic HSFD did not alter the direct vasodilatory effects of **(C)** the sGC stimulator BAY 60-4552 or **(D)** the sGC activator GSK2181236A. All data are mean ± SEM of 8–14 animals. Statistical comparisons of vasodilatory pEC_50_ and *E*_max_ values are provided in Table 3.

At the end of study, thoracic aortae were isolated and used to assess contractile function. Aortae from HSFD SHR-SP exhibited a reduced maximal relaxation response to the endothelium-dependent vasodilator carbachol as compared to tissues from ND SHR-SP (Table 3; Figure 8A). In addition, the relaxation response to the endothelium-independent vasodilator and NO-donor SNP was right-shifted in HSFD SHR-SP (Table 3; Figure 8B). Chronic treatment with GSK2181236A (0.1 and 1 mg/kg/day) or BAY 60-4552 (0.3 and 3 mg/kg/day) failed to significantly restore the attenuated endothelium-dependent and -independent vasodilation observed in HSFD SHR-SP (Table 3). Contractile responses to phenylephrine were not altered between any treatment groups (Table 3).

**Table 3:** **Effects of chronic GSK2181236A or BAY 60-4552 treatment on vasoactivity of SHR-SP isolated aorta**.

*Treatment group*	Phenylephrine		Carbachol		Sodium nitroprusside	
	EC_50_ (nM)	*E*_max_ (% KCl)	EC_50_ (nM)	*E*_max_ (% reversal of PE tone)	EC_50_ (nM)	*E*_max_ (% reversal of PE tone)
ND (*n* = 14)	67 ± 6	90 ± 4	119 ± 15	85 ± 3***	5 ± 1**	108 ± 4
HSFD (*n* = 8)	72 ± 11	83 ± 10	260 ± 74	47 ± 11	16 ± 3	103 ± 9
HSFD + BAY 60-4552 (0.3 mg/kg; *n* = 6)	52 ± 7	88 ± 6	460 ± 83	54 ± 6	25 ± 7	98 ± 5
HSFD + BAY 60-4552 (3 mg/kg; *n* = 9)	52 ± 8	89 ± 7	328 ± 41	67 ± 6	26 ± 10	112 ± 3
HSFD + GSK2181236A (0.1 mg/kg; *n* = 4)	46 ± 12	93 ± 8	407 ± 158	48 ± 14	26 ± 15	111 ± 3
HSFD + GSK2181236A (1 mg/kg; *n* = 9)	76 ± 16	91 ± 8	385 ± 89	59 ± 4	19 ± 5	100 ± 2

All values are expressed as mean ± SEM. *E*_max_ values are expressed as percent reversal of phenylephrine (PE)-induced tone except for phenylephrine where values are expressed as percent contractile response to 60 mM KCl. Statistical comparisons of both pEC_50_ and *E*_max_ values were performed using ANOVA analysis with a Dunnett’s post-test where **P < 0.01 and ***P < 0.001 vs. HSFD control SHR-SP.

As compared to ND SHR-SP, chronic HSFD did not alter the direct vasodilatory effects of the sGC stimulator BAY 60-4552 [EC_50_ 75 ± 16 nM and *E*_max_ 110 ± 2% vasodilation in ND (*n* = 15); EC_50_ 54 ± 14 nM and *E*_max_ 111 ± 2% vasodilation in HSFD (*n* = 8); Figure 8C] or the sGC activator GSK2181236A [EC_50_ 270 ± 70 nM and *E*_max_ 110 ± 3% vasodilation in ND (*n* = 15); EC_50_ 321 ± 168 nM and *E*_max_ 110 ± 7% vasodilation in HSFD (*n* = 6); Figure 8D]. These results are in contrast to the blunted vasodilatory responses seen with the NO-dependent vasodilators carbachol and SNP (Table 3; Figures 8A,B).

## Discussion

In the present study, we compared the effects of the sGC stimulator BAY 60-4552 with those of the sGC activator GSK2181236A on cardiovascular physiology in acute and chronic models of cardiovascular disease associated with oxidative stress. The main findings are: (1) in acute coronary artery I/R rats, pre-treatment with either compound did not offer cardioprotection, (2) in SHR-SP, both compounds improved survival and provided partial protection against chronic HSFD-induced end-organ damage, (3) GSK2181236A, but not BAY 60-4552, attenuated cardiac hypertrophy in a blood pressure-independent manner, and (4) the *ex vivo* vasodilatory effects of the NO-dependent vasodilators carbachol and SNP, but not the NO-independent vasodilators BAY 60-4552 or GSK2181236A, were attenuated by chronic HSFD in SHR-SP, indicating that impaired NO-bioavailability and not the oxidative state of sGC is responsible for the vascular dysfunction observed in this disease model. Together, these results indicate that the oxidation state of sGC in HSFD SHR-SP might be differentially altered between tissues. If sGC is oxidized to a greater extent in the heart compared to the vasculature, then sGC activation might be advantageous over sGC stimulation for mitigating cardiac hypertrophy associated with cardiovascular disease.

GSK2181236A was originally identified following a high throughput screen for compounds which increase the enzymatic activity of sGC. Subsequent mechanistic studies using ODQ, a sGC heme-oxidant, indicated that GSK2181236A functions as a sGC “activator” (i.e., it increases the enzyme activity of the oxidized, NO-insensitive form of sGC in a heme-independent manner). Specifically, consistent with the effects of ODQ on other sGC activators (Stasch et al., 2002b, 2006; Schindler et al., 2006; Zhou et al., 2008), ODQ pretreatment of rat vascular smooth muscle cells or vascular tissue augmented the functional effects (P-VASP formation and vasodilation, respectively) of GSK2181236A. Additional characterization of GSK2181236A in cell-based assays revealed that the interaction with sGC was at least 23-fold more potent than any other non-sGC protein tested, further supporting the utility of GSK2181236A as a pharmacological tool. BAY 60-4552, a sGC “stimulator” (i.e., it increases the enzyme activity of only the reduced, NO-sensitive form of sGC in a heme-dependent manner) currently being investigated in patients with biventricular heart failure (Mitrovic et al., 2009), was used for comparative purposes. As expected, in contrast to GSK2181236A, ODQ attenuated the functional effects of BAY 60-4552 *in vitro*.

One of the main adverse events associated with sGC stimulation/activation is hypotension (Mitrovic et al., 2009; Erdmann et al., 2010). This problem was mitigated in the current study by selecting non-hypotensive and modestly hypotensive doses of GSK2181236A and BAY 60-4552. Both doses are likely to be physiologically relevant since sGC stimulators/activators have been previously shown to elicit blood pressure-independent beneficial effects during chronic renal disease in subtotally nephrectomized rats (Benz et al., 2007) and in aged spontaneously hypertensive rats (Jones et al., 2009). Overall, both compounds elicited similar dose-dependent effects on heart rate and blood pressure, with GSK2181236A being ∼3-fold more potent. Equi-efficacious doses of both compounds were used for the I/R and SHR-SP studies.

Administration of GSK2181236A and BAY 60-4552 2 h prior to cardiac I/R failed to reduce infarct size. This was an unexpected finding considering that administration of NO-donors before cardiac I/R consistently diminishes infarct size (Bolli, 2001; Schulz et al., 2004). Although NO could exert cardioprotective effects via non-sGC/cGMP mechanisms such as direct nitrosylation of proteins (Stamler et al., 1992; Choi et al., 2002), it is widely accepted that the cGMP/PKG pathway is the pivotal mechanism by which NO mediates cardioprotection during I/R. Indeed, it has been shown that natriuretic peptides and phosphodiesterase inhibitors (which also modulate cGMP levels) reduce infarct size following I/R (Burley et al., 2007). Whereas several decades of intense investigations support a protective role for cGMP in I/R injury, the role of direct NO-independent sGC stimulation/activation during cardiac I/R is only beginning to be established. The sGC activator BAY 58-2667 improved cardiac function when administered 5 min prior to reperfusion following 60 min global cardiac ischemia in dogs (Korkmaz et al., 2009). Similarly, when administered 5 min prior to reperfusion following 30 min regional ischemia, BAY 58-2667 decreased infarct size in rat (Krieg et al., 2009) and rabbit hearts (Krieg et al., 2009; Cohen et al., 2010). Interestingly, inhibition of NO production with the eNOS inhibitor N^ω^-nitro-l-arginine methyl ester (l-NAME) exerted differential effects in these studies, blocking the cardioprotective effects BAY 58-2667 in rabbit (Cohen et al., 2010) but not in rats (Krieg et al., 2009). These results indicate a PKG-independent role for endogenous NO during I/R which could be species-dependent. In comparison to these studies where drug treatment was initiated 5 min prior to reperfusion, we administered drug 2 h prior to the ischemic insult. Additional studies are required to determine whether or not the timing of drug administration or another factor is responsible for the lack of cardioprotection observed in the present study.

Similar to previous studies, HSFD in SHR-SP induced a progressive, malignant hypertension with subsequent development of renal dysfunction (microalbuminuria), mild cardiac hypertrophy, and increased morbidity/mortality (Kerr et al., 1999; Barone et al., 2001; Behr et al., 2001; Ma et al., 2001). The gene expression profile observed in this study (increased ANF, α-skeletal actin, and TGF-β1 and decreased α-MHC expression) was consistent with maladaptive cardiac remodeling. Endothelial dysfunction, resulting from increased reactive oxygen species (ROS; e.g., superoxide and peroxynitrite, the reactive nitrogen intermediate), reduced bioavailable NO, or impaired NO-induced activation of oxidized sGC, contributes to the development and progression of this chronic hypertension and is likely a responsible mechanism for the hypertension-induced end-organ damage in this model (McIntyre et al., 1997, 1999; Ma et al., 2001; Ju et al., 2003; Stasch et al., 2006).

Considering that the vasorelaxation and cardiac and renal protective effects of the sGC activator BAY 58-2667 were potentiated under pathophysiological and oxidative stress conditions (Stasch et al., 2006) and that removal of the sGC heme moiety or its inactivation by oxidation strongly diminished the ability of sGC stimulators to modulate sGC (Evgenov et al., 2006), it has been hypothesized that pharmacological enhancement of the NO/sGC pathway via sGC activators, not stimulators, would be the more effective therapy for prevention and treatment of cardiovascular disease in the presence of excess ROS. This hypothesis has not previously been tested comprehensively *in vivo*. In contrast to expectations, however, improvement of endothelial function was not potentiated by sGC activation as compared to sGC stimulation. Instead, the vasorelaxation, blood pressure lowering, and renal protective effects of the sGC stimulator BAY 60-4552 were similar to or slighter greater than that of the sGC activator, GSK2181236A, at the doses examined in this study. Overall, these results suggest that the oxidation state of sGC capable of modulating blood pressure and renal function is not altered to an extent necessary to exploit the differing mechanisms of actions of these pharmacological tools.

Interestingly, however, GSK2181236A, but not BAY 60-4552, attenuated cardiac hypertrophy in a blood pressure-independent fashion. These results suggest that sGC in cardiac tissue might be oxidized to a level sufficient to distinguish between sGC stimulation and activation. Growing evidence suggests that ROS play a pathophysiological role in the development of cardiac hypertrophy (see Seddon et al., 2007 for review), and indicates that sGC activation might be advantageous over sGC stimulation for mitigating maladaptive cardiac hypertrophy associated oxidative stress. However, additional studies are required to elucidate regional differences in sGC oxidation state.

The vasodilatory responses to compounds which modulate the NO/sGC pathway at different points were investigated in HSFD SHR-SP to help elucidate the mechanism(s) responsible for the vascular dysfunction. The vasodilatory responses to the endothelium-dependent vasodilator carbachol and the endothelium-independent vasodilator SNP were attenuated by HSFD, consistent with previous observations (Ju et al., 2003; Willette et al., 2009). These results alone could be explained by multiple impairments in the NO/sGC pathway, including reduced NO production, excessive NO degradation/neutralization, sGC oxidation, altered phosphodiesterase activity, etc., and thus do not clarify the mechanism for the impaired vasodilation. In contrast to NO-dependent vasodilation, however, the vasodilatory responses to the NO-independent compounds BAY 60-4552 and GSK2181236A were unaltered by HSFD. Taken together, three important conclusions can be made. First, the mechanism responsible for the impaired vasodilation is upstream of sGC and is likely reduced NO-bioavailability due to the high levels of oxidative stress and subsequent peroxynitrite formation associated with HSFD in SHR-SP (Ma et al., 2001). Second, the level of oxidative stress in this model is high enough to inactivate NO, but not high enough to alter the oxidation state of sGC to a point necessary to impact the vasodilatory effects of direct (NO-independent) sGC stimulators or activators. Rather, a level of sGC oxidation seen with 10 μM ODQ is likely necessary to impact NO-independent sGC stimulator/activator-mediated vasodilation. As such, it is questionable whether or not the ODQ results represent a biochemical artifact or a pathophysiological state. Interestingly, in contrast to our results with GSK2181236A, Stasch et al. (2006) have shown that the vasodilatory effects of a different sGC activator, BAY 58-2667, are potentiated under pathophysiological conditions in animal models [aged SHR, hyperlipidemic (WHHL) rabbits and atherosclerotic (*ApoEl*^−/−^) mice] and humans (mesocolon arteries taken from patients with type 2 diabetes). The precise reason for this discrepancy is unknown, but could be the result of different animal models or compounds. Indeed, although both GSK2181236A and BAY 58-2667 are sGC activators with structural similarities, their precise mechanism(s) of action might differ. Additional studies are necessary to elucidate these discrepancies. Third, consistent with the *in vivo* hemodynamic findings, the hypothesis that sGC activation offers a therapeutic advantage over sGC stimulation is not necessarily correct. Although this hypothesis appears accurate when comparing NO-mediated stimulation of sGC (e.g., via muscarinic receptor activation or NO-donors) with sGC activators (e.g., BAY 58-2667; Stasch et al., 2006, GSK2181236A), other than differential effects on cardiac hypertrophy, there was no clear differentiation between NO-independent sGC stimulation and activation in the present study. These findings emphasize that additional studies which directly compare NO-independent sGC stimulators with activators are necessary in order to better predict which class of agents might offer a therapeutic advantage in a particular patient population.

In summary, the present study is the first to compare a NO-independent sGC stimulator (BAY 60-4552) with a sGC activator (GSK2181236A) directly and comprehensively both *in vitro* and *in vivo*. Whereas neither compound attenuated acute cardiac I/R injury, both provided dose-dependent levels of protection against HSFD-induced end-organ damage in SHR-SP, a model associated with profound oxidative stress, hypertension, and renal dysfunction. Isolated artery dilation studies indicate that reduced NO-bioavailability and not the oxidation state of sGC is responsible for the HSFD-induced impaired vasodilation, and thus provide a rationale as to why the hypotensive effects of sGC activation was not potentiated. In contrast, the differential effects of sGC stimulation and activation on HSFD-induced cardiac hypertrophy suggest that the oxidative state of sGC is altered to a greater degree in cardiac than vascular tissue. Overall, these results suggest that the vasorelaxation, blood pressure, and end-organ protective effects of sGC activators can be clearly differentiated from those of NO-donors, but not from those of sGC stimulators in rodent models of oxidative stress. However, sGC activation might be advantageous over sGC stimulation for mitigating cardiac hypertrophy associated with cardiovascular disease. Additional studies designed to further define the pharmacological differences between sGC stimulators and sGC activators will be necessary to understand the potential clinical differentiation of these two classes of agents.

## Conflict of Interest Statement

All authors are/were employees of GlaxoSmithKline and own company stock.

## Abbreviations

ANF, atrial natriuretic factor; BNP, brain natriuretic peptide; cGMP, cyclic guanosine 3′,5′-monophosphate; DMSO, dimethylsulfoxide; HPMC, hydroxylpropyl methylcellulose; HSFD, high salt/fat diet; IM, intramuscular; IP, intraperitoneal; I/R, ischemia/reperfusion; KHB, Krebs–Henseleit buffer; L-NAME, N^ω^-nitro-L-arginine methyl ester; MALB, microalbumin; MAP, mean arterial pressure; ND, normal diet; NO, nitric oxide; ODQ, 1H-[1,2,4]oxadiazolo[4,3-a]quinoxalin-1-one; PBS, phosphate-buffered saline; PKG, protein kinase G; ROS, reactive oxygen species; sGC, soluble guanylate cyclase; SHR-SP, spontaneously hypertensive stroke prone rat; SNP, sodium nitroprusside; SQ, subcutaneous; (P)-VASP, (phosphorylated) vasodilator-stimulated protein.
